# The effect of anastomotic leak on postoperative pelvic function and quality of life in rectal cancer patients

**DOI:** 10.1007/s12672-022-00518-w

**Published:** 2022-06-25

**Authors:** Aris Plastiras, Dimitrios Korkolis, Maximos Frountzas, George Theodoropoulos

**Affiliations:** 1 Department of Surgical Oncology, St Savvas Oncologic Centre of Athens, Athens, Greece; 2 Colorectal Unit, First Department of Propaedeutic Surgery, Medical School of National and Kapodistrian University of Athens, Hippocration Hospital, 114 Vas Sofias Ave, 11527 Athens, Greece

**Keywords:** Rectal cancer, Anastomotic leak, Pelvic function, Sexual function, Quality of life

## Abstract

**Aim:**

The aim of this review was to collect all available literature data analysing the effects of the anastomotic leak (AL) on post-sphincter preserving rectal cancer surgery bowel and urogenital function as well as to quality of life (QoL) dimensions.

**Methods:**

A literature search of the PubMed and Embase electronic databases was conducted by two independent investigators and all studies using either functional parameters or QoL as a primary or secondary endpoint after a rectal cancer surgery AL were included.

**Results:**

Amongst the 13 identified studies focusing on the post-AL neorecto-anal function, 3 case-matched studies,3 comparative studies and 1 population-based study supported the deleterious effects of the AL on bowel function, with disturbances of the types of high bowel movement frequency, urgency and increased incontinent episodes to predominate. At one case-matched study the Low Anterior Resection Syndrome (LARS) score was inferior in the AL patients. At limited under-powered studies, urinary frequency, reduced male sexual activity and female dyspareunia may be linked to a prior AL. According to two QoL-targeted detailed studies, QoL disturbances, such as physical and emotional function difficulties may persist up to 3 years after the AL occurrence.

**Conclusions:**

AL may have adverse effects on postoperative pelvic function and QoL in rectal cancer patients. As evidenced by this literature review, the limited reports on this intriguing topic may trigger the initiative for planning and undertaking larger, multicentre studies on rectal cancer patients with varying degrees of AL severity.

## Introduction

Surgery for colorectal pathologies is plagued by the frequently encountered inadvertent occurrence of an anastomotic leak (AL) [1]. In general, roughly one out of ten rectal cancer patients may experience this morbid and potentially lethal complication, whereas colorectal surgeons may face therapeutic strategies dilemmas and may need to employ a variety of techniques to ensure preservation of the jeopardised bowel continuity. Not only the devastating consequences of AL are reflected by the sharp increase of the short-term postoperative morbidity and mortality, but also by the associated inferior long-term oncologic outcomes and decreased cancer-related survival [2].

Optimal functional outcomes in the sense of satisfactory bowel evacuation and anorectal function and preservation of urinary and sexual function, as well as restoration of the basic quality of life (QoL) domains have all been established as important outcome measures of surgical treatment success and may be closely related and directly affected by anatomic and physiologic disturbances arising from a postoperative complication, such as an anastomotic failure. Rather limited data exist in the literature focusing on assessment of defecatory function, urogenital function and QoL after an AL at rectal cancer patients.

This review focuses on the various assessment attempts conducted up to now, and aimed at analyzing the potentially negative effects of AL on functional parameters after colorectal cancer surgery. Our scope was to collect existing knowledge on functional sequelae related to an undesirable AL, to summarize important points and to present them in a comprehensive manner.

## Methods

This review was conducted according to PRISMA (Preferred Reporting Items for Systematic Reviews and Meta-analysis) guidelines. Two independent investigators (A.P. and M.F.) searched PubMed/Medline and Embase electronic databases using the following as key words: “rectal cancer” OR “total mesorectal excision” AND “anastomotic leak”, AND “functional outcomes”, “bowel function”, “urinary function”, “sexual function”, “urogenital function”, AND “health-related quality of life”, “quality of life”. Articles meeting these searching criteria, written in English and published after 1996, were included. Any controversies were resolved by a third, independent investigator (D.K.)

**Fig. 1:**
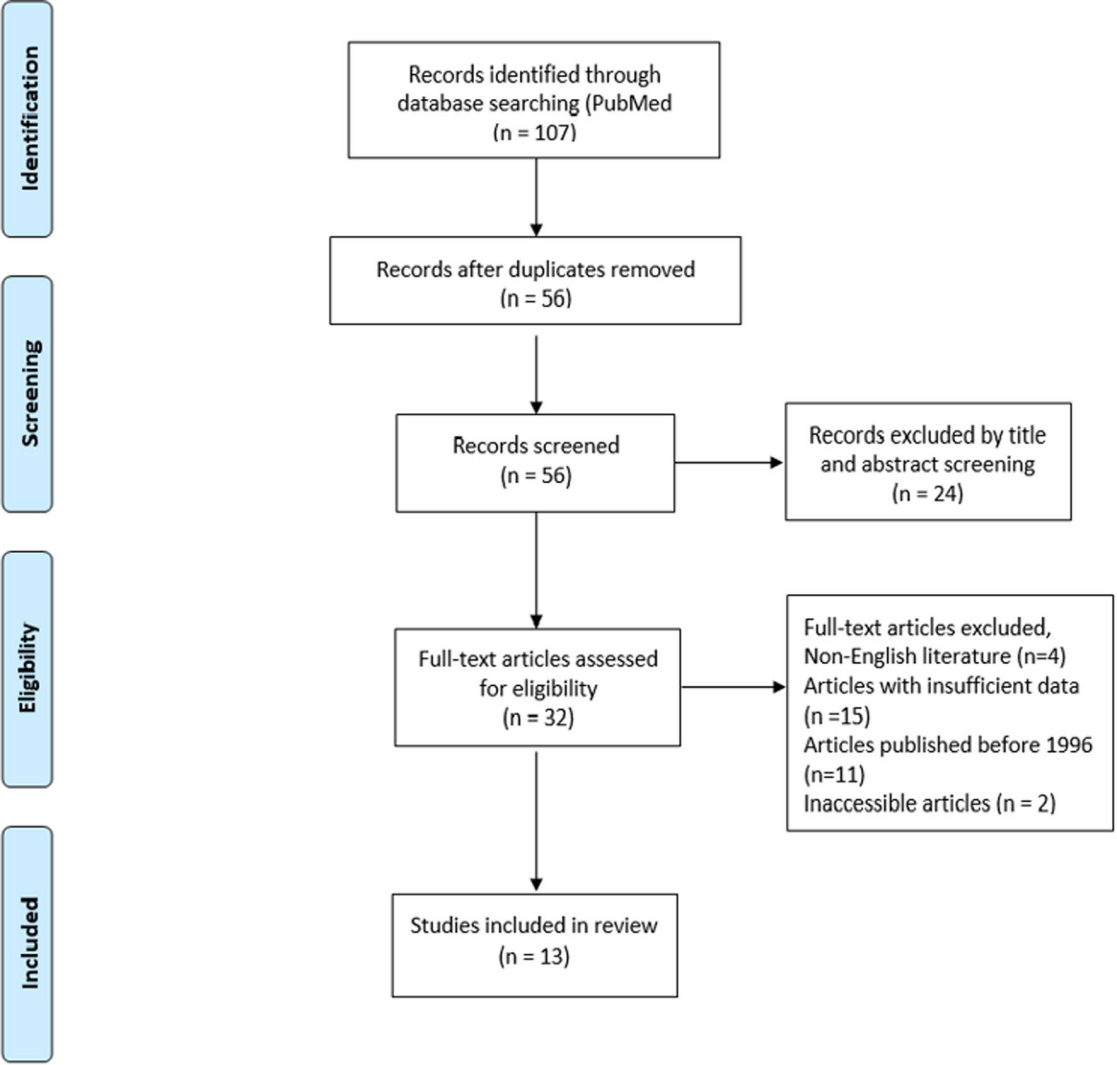
Flow chart of the studies

Out of the 107 studies identified through database searching, 32 were identified to be eligible for as they used functional parameters or QOL as primary or secondary endpoints after colorectal surgery. Articles with insufficient data, not written in English or with inaccessible full text were excluded. Amongst the eligible studies, only 13 studies were included in our review as they focused on either functional or QoL alterations following rectal cancer surgery AL (Fig. 1).

The methodological quality of studies, which all occurred to be non-randomized, was assessed with a MINORS score [2]. Since the number of existing studies was rather limited, no specific threshold was used as pre-requirement for final inclusion, but a score of ≥ 14 denoted an adequate study quality.

## Results

Studies features and main features are summarized Amongst the 13 identified studies [3–15], 6 case matched studies [3, 4, 7, 9, 10, 15] compared a total of 138 AL patients to 227 non-AL controls. The authors of 6 non-randomised retrospective comparative studies [5, 6, 8, 12–14] investigated functional sequelae of 289 AL patients compared to 2,514 patients after uneventful rectal cancer surgery. All studies defined AL and included symptomatic patients with clinically evident AL, whereas 2 studies [6, 9, 10] added patients with subclinical, radiologically evident ALs. Although AL severity grading was mentioned in only 2 studies [5, 15], management of the AL patients specifically included in the studied AL groups was clearly stated at 9 studies [3–7, 10–12, 15]. Collectively of the total of 181 AL patients, 42 (23.2%) required relaparotomy, while 70 (38.6%) were managed by drainage (either transrectal or CT-guided). In 49 of the 80 (61.2%) of the AL patients included as one of the groups at 5 studies revealing such an information, an initial protective loop stoma had been constructed [3–6, 15].

In 7 studies [3, 4, 8–10, 12, 14] self-constructed questionnaires focusing on patients’ evacuation habits were used. Validated scoring systems and their respective scales, either as an addition or as a sole means of evaluation, were used to estimate bowel functional parameters at 8 of the studies [5–7, 9–13], whereas only 1 of the 2 studies focusing on urogenital dysfunction was based on universally accepted questionnaires [7]. At all 7 QoL studies [6–10, 13, 15] validated questionnaires were applied. Relative details on specific questionnaires used at each study are reported at the results’ subsections of this review (Table1).

**Table 1:** Studies characteristics and main outcomes

Author [ref.]	Year	Study type	MINORS scale	AL/non-AL patients	Follow-up	Outcomes (bowel and urogenital function)	Conclusions	Outcomes on QoL
Hallböök and Sjödahl [3]	1996	CM	14	19/19	30 mos (12–87)	Sphincter function maintained Reduced neorectal volume, increased frequency and urgency of bowel movements	Bowel function compromised	
Nesbakken et al. [4]	2001	CM	14	11/11	12–48 mos	Sphincter function maintained Reduced MTV	Bowel function compromised	
Bittorf et al. [5]	2003	RC	15	22/128	107 ± 46 wks	AL with less incontinence No significant difference in urgency or MTV, or patient satisfaction	Bowel function undisturbed	
Lim et al. [6]	2006	RC	16	23/75	10–18 mos	Bowel function was worse at both subclinical and clinical AL patients compared to non-AL patients	Bowel function compromised	Μedian QoL scores were similar at between the non-AL and the AL (either clinical or subclinical) patients Worst QoL scores were observed et al. patients who still had their ileostomy
Riss et al. [7]	2011	CM	13	16/16	106.8 mos (32.4–170.4)	No significant differences at Vazey and Wexner constipation score	Bowel function undisturbed Urogenital function undisturbed	Physical and mental components were not significantly different between the AL and non-AL patients
Ashburn et al. [8]	2013	RC	18	52/812	3.3 yrs	Increased frequency and incontinence/ pad use at 1 y QoL compromised at 1 year and most recent follow-up (3.2 yrs)	Bowel function compromised	Physical and mental components were significantly worse et al. patients up to 1 yr and up to 3 years, respectively, after surgery
Mongin et al. [9]	2014	CM	14	21/42	30 mos	No difference at frequency of bowel movements, stool fragmentation, ability to defer defection and Wexner incontinence score. Use of pads was significantly more frequent in AL patients	Bowel function undisturbed	Physical function subscale was negatively affected in the AL compared to the non-AL group of patients Lifestyle, coping/behaviour, depression/self-perception, and embarrassment FIQL scores were all reduced at the AL patients
Hain et al. [10]	2017	CM	14	46/89	46 ± 26 mos	AL patients had impaired LARS score. No difference between asymptomatic AL group and uncomplicated patients AL compared to the patients with symptomatic AL reported frequent urination per day	Bowel function compromised Urinary function compromised	
Hughes et al. [11]	2017	Cohort	13	5	248 days	Higher non-significant incidence of AL within the major LARS cohort	Bowel function compromised	
Yokota et al. [12]	2017	RC	17	46/292	2 yrs	AL group worse with difficulties in evacuation, discrimination between stool and flatus, and night-time soiling, but not at stool frequency, daytime soiling, lifestyle alteration, and need for anti-diarrheal medication. Worse Wexner incontinence score at anastomotic dehiscence group	Bowel function compromised	
Miura et al. [13]	2017	RC	12	27/116	63 mos	No difference at frequency of defecation per day and Wexner scores	Bowel function undisturbed	No significant difference between the FIQL scores of the AL and the non-AL patients
Hultberg et al. [14]	2020	RC	17	89/1091	2 yrs	Increased risk of aid use for fecal incontinence after AL and trend toward increased defecation frequency. Risk of fecal incontinence and defecation at night similar between groups Risk of urinary incontinence was non-significantly decreased in the AL patients. The risk of voiding difficulties was similar between AL and non-AL patients. Among women, the risk of dyspareunia after AL was significantly increased. Among men, no difference in erectile or ejaculation dysfunction but with reduced sexual activity after AL	Bowel function compromised Urinary function undisturbed Sexual function compromised	
Marinatou et al. [15]	2014	PCM	18	25/50	1 yr	Bowel function compromised		3 mos after surgery: worse “physical function” and “stoma related problems” et al. patients 6 and 12 mos after surgery: worse physical, emotional, social function, role limitations and general health et al. patients

*CM* case-matched, *RC* retrospective comparative, *mos* months, *wks* weeks, *yrs* years, *MTV* maximal tolerable volume, *QoL* quality of life, *FIQL* fecal incontinence quality of life

### Bowel function

The first attempt to elucidate the clinical and physiologic effects of AL on a colorectal anastomosis was in a Swedish case-matched study where 19 rectal cancer patients with AL were compared with an equal number of patients without AL [3]. The anorectal function was clinically evaluated by a composite questionnaire assessing the daily frequency of bowel movements, grade and regularity of fecal incontinence, the capability to delay defecation and also the degree of impaired evacuation. The study was complemented by manovolumetric measurements of neo-rectal compliance and sensory thresholds. At a follow-up of 30 months, although there was no difference in sphincter function between the arms, the neorectal volume at distension pressures of 40 and 50 cmH_2_O was considerably lower in AL group. The compliance values at sensation of repletion, urge to open bowels and maximum tolerated amount of stools were significantly lower in the leakage group. The impaired AL patients’ function was indicated by the significantly higher score of frequency, urgency, incontinence and impaired defaecation, compared to the non-AL patients. Frequency of stools was significantly higher in leakage group but the increase of urgency and incontinence score did not reach statistical significance between the compared groups [3].

In a small comparative study, Nesbakken et. al. explored the functional outcome of 11 post-AL rectal cancer patients of whom the stoma had been reversed and the bowel continuity had been uneventfully restored without stricture with matched patients who had not experienced AL [4]. Although anal sphincter pressures were not different between the compared arms, patients with a history of AL had significantly lower maximum tolerable neorectal volumes than controls 1–2 years after bowel function reinstatement. At the same follow-up period, the functional disorder that most AL patients suffered was the inability for complete bowel evacuation. A trend for a higher degree of fecal urgency was demonstrated in the AL group. Only few patients reported occasional incontinence to gas and loose stools, but the difference between AL and non-AL patients did not reach statistical significance [4].

In a later German study, 22 rectal cancer patients with a confirmed AL both clinically and radiologically were compared to 128 patients with an uneventful postoperative course, who had been managed at the same institution [5]. Continence as assessed by the Cleveland Clinic Continence Score (CCCS) (Wexner’s score) was slightly better at the AL patients, while the rest of clinical parameters denoting evacuation dysfunction, such as soiling, urgency and sampling between solid and flatus were slightly better at the non-AL group [16]. Nonetheless, evacuation was superior amongst the AL patients. Overall satisfaction with bowel function was reached high levels of more than 80%, without statistically significant differences between the patients’ groups. Anew, no significant differences were observed at the manovolumetric parameters, even though non-significant better anal pressures and worse average maximum tolerable volume and neorectal compliance were revealed after AL [5].

Lim et. al. followed-up 138 consecutive rectal cancer patients and they identified 13 clinically presented AL and 10 subclinical, radiologically apparent AL [6]. Although all subclinical ALs were followed by a successful protective ileostomy closure, only 30% of the clinical ALs had their stoma reversed. Patients with intestinal continuity restored were asked to fill a validated bowel function (BF) questionnaire, whose scores ranged from a minimum of 13 to a maximum of 45, with higher scores reflecting poorer bowel function. At an average follow-up of about 2 years, bowel function was worse at both subclinical and clinical AL patients compared to non-AL patients, but this difference reached statistical significance only for the subclinical AL patients compared to uncomplicated patients [6].

In an Austrian study, Riss et. al. matched 16 available AL patients with an equal number of non-AL patients used as a control and they investigated defecatory dysfunction at a median follow-up of 106.8 months (32.4–170.4) [7]. They used the Vazey Incontinence Score and the Wexner Constipation score to evaluate the respective bowel disturbances [17, 18]. They did not discover any significant difference and the Vazey median score was 8 (range 0–24) in the patient group and 5 (0–16) in the control group. Similarly, the median Wexner Constipation score of the control group was non-significantly, slightly lower compared to the patients’ group [2 (0–12) vs. 5 (0–16)] [7].

In a single-institution study amongst 864 rectal cancer patients surgically managed over a 24-year period at the Cleveland Clinic Foundation (CCF), 52 patients (6%) were presented with a postoperative AL [8]. Patients with AL were younger, more likely to be male, more likely to have undergone proximal diversion at the time of proctectomy, while they had more distal rectal tumours and required lower levelled anastomosis. One year post surgery, AL patients had significantly more daytime and night-time bowel movements, and worse control of solid stool compared to those without AL. Most recent follow up (AL, 3.3 years vs no AL, 2.4 years) showed that patients with an anastomotic leak reported worse mental component scores and increased use of perineal pads. [8].

The authors of a French study compared 21 patients with AL after laparoscopic total mesorectal excision (TME) with 42 patients without AL and they assessed them at the end of the most recent follow-up (average 30 months for the Al and 33 months for the non-Al patients, range: 6–75 months) [9]. Frequency of bowel movements was not different between the two groups, either on daytime or night-time. Stool fragmentation and lack of capability to defer defaecation for 15 min were frequent but without any difference, comparing patients when anastomotic leak is present or not. Pads use was significantly more frequent in AL group. However, overall Wexner’s score did not reach any significant difference between the compared arms. Authors concluded that patients with AL should be informed that if they initially experience severe symptoms, outcomes tend to be overtime similar to uncomplicated patients [9].

Another French case-matched study was the first that utilise well validated Low Anterior Resection Syndrome (LARS) score to evaluate long-term bowel dysfunction associated with AL [10, 19]. From a tertiary referral colorectal centre prospectively maintained database, patients with postoperative AL (23 with symptoms and 23 with no symptoms) after laparoscopic, sphincter-saving surgery for rectal cancer were compared to 89 non-AL matched patients. Following-up of 46 months, AL patients experienced impaired LARS score (median values: 30 vs 27 in control group (p = 0.02). No LARS was detected in 4% of AL patients (vs 31%), minor LARS in 52% (vs 52%), and major Low Anterior Resection Syndrome in 44% (vs 17%) (p = 0.004). Comparing asymptomatic AL group and the uncomplicated patients’ group showed no difference for median LARS score and LARS categories [10].

Patients’ analysis included in the LARRIS Trial demonstrated a high incidence of major LARS (56%) amongst the 68 post-surgery rectal cancer responders [11]. Five patients (7%) had developed AL within the postoperative period. Out of five anastomotic leak patients, four of them reported major LARS symptoms. Despite the fact that a higher incidence of AL was noticed in the major LARS cohort, this did not reach statistical significance in the multivariable analysis [11].

In a Japanese study, Yokota et. al. classified 341 patients into three groups after intersphincteric resection for rectal cancer: anastomotic dehiscence (AD), major AL (Clavien–Dindo grade III +), or control (< grade III or no AL) groups [12]. They repetitively assessed their functional status throughout the first 2 postoperative years and, having a 70% response rate, they found that the major AL group had a Wexner score equal to that of the control group at 2 year follow-up whilst the AD group remained with high Wexner scores, even at this late follow-up assessment point [16]. Actually, the amount of patients with a high Wexner score (> 16) at the AD patients (40%) was twice the one of the rest of the patients (20%). Although continence of the major AL patients was quite close to control group, the AD group experienced worse liquid stools and gas continence compared to the other groups [16]. The major AL group also tended to fare worse than the control group in terms of difficulties in evacuation, discrimination between stool and flatus, and night-time soiling, but not at stool frequency, daytime soiling, lifestyle alteration, and need for anti-diarrheal medication over the 2-year period [12].

In another Japanese study, Miura et. al. aimed at evaluating permanent stoma and evacuating function in long-term follow up after low rectal cancer surgery without diverting stoma [13]. Defecation function was assessed by mailing a questionnaire to patients who developed AL (n = 27, response rate: 45.0%) and 116 patients who did not develop AL (response rate: 53.9%). In a follow-up of 63 months, frequency of defecation per day (median (quartile): 4 (1.5–4)] and Wexner scores 6 (2.5–9) at the AL responders were favourably compared with the non-AL responders [median quartile: 4 (1.5–4)] [13].

In the most recent retrospective population-based cohort study, rectal cancer patients subjected to LAR at a 3-year period were identified through the Swedish Colorectal Cancer Registry Functional outcomes were assessed after surgery (2 years) via postal questionnaire [14]**.** Response rate was 82%, resulting in 1180 included patients; AL identified in 7.5% of patients. Permanent stoma was more likely among patients with AL (44% vs 9%; *p* < 0.001). An increased need of aid use for incontinence (ex, panty liners, sanitary pads, or nappies/diapers) after AL (OR, 2.27; 95% CI, 1.20–4.30) and a trend toward increased defecation frequency (1.06; 95% CI, –0.04 to 2.17) was noted, but at the same risk of fecal incontinence and defecation at night did not differ between groups [14].

### Urogenital dysfunction

Regarding to the first of the three existing studies dealing with the potential sequelae of AL on urinary and/or sexual function, Riss et. al. used the “International Consultation on Incontinence Questionnaire-Short Form” (ICIQ-SF) (no urinary incontinence: 0 points, severe urinary incontinence 21 points) and the validated “International Prostatic Symptom Score” (I-PSS), which contains 7 questions (no symptoms: 0 points, maximum symptom score: 35 points), the validated “Female Sexual Function Index” (FSFI), and the German version of the “International Index of Erectile Function” (IIEF) (a higher score denotes better sexual function) [7, 20, 21]. Based on ICIQ-SF, the control group registered with a significantly lower score compared to the patient group [median: 0.5 (0–10) vs 0 (0–8)] (p = 0.04). Three patients complained about stress incontinence and 4 patients of urge incontinence. Contrastingly, 2 control subjects reported urge incontinence and 1 complained about mixed incontinence. Interestingly, no difference was detected when groups were compared according to the I-PSS. IIEF scoring did not reveal any significant difference at the male sexual function between AL and non-AL patients [median: 39 (range 9–61) vs. 32.5 (range: 12–57)]. The FSFI could not offer information due to the under-representation of females [7].

Based on the respective urinary functional domains of the European Organization for Research and Treatment of Cancer (EORTC) QLQ CR-29 validated questionnaire, Hain et. al. found that the combined groups of patients with no or asymptomatic leakage compared to the patients with symptomatic AL suffered frequent urination per day (score: 33 [range: 0–100] vs 50 [range: 0–83], p = 0.03) [7, 22].

In the most recent published study, Hultberg et. al. used a Swedish professional translation of the questionnaire in the Dutch total mesorectal excision trial that consisted 20 questions concerning pre-and postoperative urinary, sexual and defecatory function [14]. AL decreased the risk of urinary incontinence (OR, 0.53; 95% CI, 0.31–0.90) and the risk of aid use for urinary incontinence (OR, 0.41; 95% CI, 0.18–0.92). The risk of the incontinence subtypes (stress/urge/combined) was non-significantly declined in the AL patients. The risk of voiding difficulties did not differ between AL and non-AL patients [14]. Among women, the risk of dyspareunia after AL was statistically significantly increased but the incidence of coital vaginal dryness was similar in both groups [14]. Among men, no significant difference in either erectile or ejaculation dysfunction was revealed between compared groups. On the other hand, the frequency of reduced sexual activity after surgery was statistically significantly increased after AL (90% vs 82%; p = 0.003) [14].

### Quality of life

Amongst the aforementioned studies, few have also focused on alterations of postoperative health-related QoL, its related domains and functional parameters, as this was assessed by validated QoL questionnaires [6–9, 15]. Lim et. al. required patients to fill the validated EORTC QoL questionnaire, at which higher scores reflected better QoL [6, 23, 24]. Amongst the 92 of 112 (83%) respondents the median QoL scores were similar at an average of 1 year after surgery between the non-AL and the AL (either clinical or subclinical) patients. Nevertheless, the worst QoL scores were observed et al. patients who still had their ileostomy (roughly QoL score of 10–20 vs 60–80 for the rest of patients, p = 0.03) [6].

Riss et. al. measured quality of life using the Short Form-12 Health Survey (SF-12) at the time of follow-up which averaged about 9 years after surgery [8, 25]. Physical and mental components (PCS and MCS) were constructed from SF-12 scoring [8, 25]. Neither the PCS nor the MCS scores were significantly different between the studied groups of this small sample of 16 patients [7].

According to the CCF data, AL patients had worse PCS at the 6^th^ and the 12^th^ postoperative month (p = 0.01) [8]. Significant MCS reduction was also observed at 6-month, 1-year, and 3-year assessments et al. patients (p = 0.01, 0.01, 0.02). PCS but not MCS scores were equivalent at the most recent patients’ follow-up, which exceeded 2 years for both AL and non-AL patients [8].

QoL outcomes were assessed by the Short Form 36 (SF-36), Faecal Incontinence Quality of Life (FIQL) and the EORTC QLQ CR-29 scores at a median follow-up of 30 months on the study conducted by Mongin et. al. [9, 26, 27]. Only the physical function subscale was negatively affected in the AL compared to the non-AL group of patients (p = 0.04), whilst the rest of subscales, as well as the PMS and the MCS scores were not impaired at the AL patients [9]. Lifestyle, coping/behaviour, depression/self-perception, and embarrassment FIQL scores were all reduced at the AL patients, with only “depression/self-perception” scale score to be significantly decreased at the AL compared to the non-AL patients; groups (p = 0.03) [9]. As for the EORTC QLQ CR29 domains, no significant difference was demonstrated at the compared groups, except for the “blood and mucus in stool” score which was significantly impaired at the AL patients (p = 0.001) [9]. Miura et. al., using a modified FIQL questionnaire at a median follow-up of about 5 years, failed to demonstrate any significant difference between the respective scores of the AL and the non-AL patients [13, 28].

A detailed documentation of postoperative QoL shifts was reported by our group, in the context of a case-matched study, conducted at our Greek academic colorectal unit, when 25 LAR patients having had experienced an AL were compared with 50 patients with an uncomplicated course [15]. QoL data were prospectively collected at fixed assessment time-points (baseline, 3, 6, and 12 months postoperatively) using the following questionnaires: SF-36, Gastrointestinal Quality of Life Index (GIQLI), EORT QLQ C30 and EORTC QLQ CR29 [22, 24, 26, 29]. At the third postoperative month of assessment, AL patients had worse “physical function” as measured by both GIQLI and EORTC QLQ-C30 questionnaires (p = 0.03). “Abdominal and pelvic pain”, “stoma-related problems” as well as “sore skin” appeared more frequently 3 months after surgery in the AL patients (p = 0.03), according to the EORTC QLQ-CR29 [22]. At the 6-months assessment the SF-36 subordinate scales “role limitations secondary to physical health”, “role limitations secondary to emotional problems”, “social function” and “general health” were significantly impaired in the AL patients (p < 0.03). GIQLI “global QoL”, “emotional function” and “physical function” were also significantly deteriorated in the AL patients (p < 0.01). “Physical functioning” and “overall QoL” as evaluated by the EORTC QLQ-30 were also worse at the AL patients at 6 months (p < 0.005), whilst the EORTC QLQ-CR29 domains of “stoma problems” and “sore skin” persisted at 6 months in the AL patients (p = 0.03). At the end of the first postoperative year, the SF-36 subscales “physical function”, “role limitations due to physical health”, “role limitations due to emotional problems”, “social function” and “general health”, the GIQLI “global” and “emotional function” scores, the EORTC QLQ-C30 “overall QoL” score and the EORTC QLQ-CR29 “sore skin” score were at a significantly worse level at the AL compared to the non-AL patients [22].

## Discussion

The distortion of large bowel anatomy after surgical resections and the impact of adjuvant therapy for colorectal cancer may lead to gastrointestinal dysfunction [30]. Rectal cancer patients appear to be more vulnerable, mainly due to rectal reservoir loss [30]. Functional restoration and preservation have been incorporated amongst the goals of sphincter-preserving surgery for cancer. The wide adoption of total mesorectal excision (TME) and the decrease of the ontologically safe distal margins have increased the risk of ALs from the low-lying “vulnerable” and technically demanding to construct colo-(rectal)anal anastomosis [15]. Beyond its well-studied negative effects on the short- and long-term recovery and oncological outcome, an anastomotic insufficiency may cause significant postoperative derangement in terms of various QoL parameters, such as bowel and pelvic function. Nevertheless, as revealed in this review, there is still lack of robust literature data establishing an undisputable link between post-sphincter preserving TME ALs and functional outcomes [9]. AL-driven pelvic sepsis and failure of healing by first intention may lead to granulation tissue formation and peri-anastomotic fibrosis, as well as anastomotic stricture and reduction of the neorectal reservoir function [3, 4]. Routine diverting ileostomy as a common practice in all low rectal anastomoses seems to be supported by many surgeons although a recent meta-analysis from UK highlights the importance of other than anastomotic leak complications in the ileostomy group which should always be considered [31]. Same results are highlighted in the RALAR multicentre study from 24 Italian referral centers. Despite the fact that ileostomy did not affect risk of AL, it does prevent from their disastrous septic outcomes and need of reoperation [32]. Answering the question whether defunctioning ileostomy should be closed within 1 month or 60 days later a recent systematic review and meta-analysis indicated some advantages of early reversal regarding less small bowel obstruction and lower rate stoma site complications [33].

The results of the published studies, though, are not uniformly in keeping with an expected deleterious effect of the AL at the patients’ postoperative anorectal function. Published studies by Bittorf et al., Riss et al., Miura et al., and Hughes et al., did not succeed to verify such a significant deranged functional occurrence compared to the uncomplicated patients’ cohorts [5, 7, 11, 13]. Those were unmatched studies with their expected limitations. The majority of case-matched studies, namely the ones by Hallbook et al., Nesbakken et al. and Hain et al. supported the negative effect of the AL on bowel function, whilst the case-matched study by Mongin et. al. did not agree with such an outcome [3, 4, 9, 10]. A principal drawback on the rather unusual studies focusing on complications, such as the AL, is the limited number of patients included, due to the frequency of the occurrence of such a mishap and the subsequent need of targeting on large patients’ populations to explore its real effects. Case matched methodology is the one best suited when exploring the consequences of uncommon complications such as AL, which, though, is difficult to numerically reach such a level for randomization of comparative studies to be feasible. Nevertheless the selected articles quality as assessed by MINORS scale reached to a satisfactory level.

In the study by Ashburn et. al, where patients from a rather numerically satisfactory sample derived from a tertiary care USA institution were assessed, and in the study by Hultberg et al., on which a nationwide including patients register, the negative sequelae of an AL was affirmed [8, 14]. In general, disturbances in types of high bowel movement frequency, urgency and increased incontinent episodes may predominate [8, 14]. Hultberg et. al. also conducted the first population-based study on this important research topic, which unavailingly leads to selection bias alleviation [14]. The high response rate of this study was an important advantage for the accuracy of their results. In such uncontrolled retrospective studies, response rates may have a serious impact on the researchers’ ability to reach valid conclusions in regards to the existence of a correlation between AL and functional outcomes.

Another important issue of studies focusing on conditions such as AL is the heterogeneity of patients themselves. This is based on the complication diversity in regard to its presentation, its severity, and its management [30]. The physiologic and functional impact may differ in patients who required just to keep the drain and the ileostomy to be closed at a later time and the ones who had to be operated on an emergent basis [30]. Early ALs are more prone to be symptomatic and require re-intervention than late ones and might acquire a more profound functional effect [14]. Only one study has attempted a severance according to the severity of AL [12]. They pointed out that the presence of dehiscence at the anastomosis site is an important factor pertaining to the persistence of severe bowel dysfunction at the long term. Conversely, even patients with a major leak may experience recovery of their poor anal function over the first 2 years after an intersphincteric resection [12]. Nevertheless, they suggested that even patients after major AL require a long period follow-up of their functional status [12].

It has to be noted that the accurate, worldwide validated, brief and clinically applicable.

LARS questionnaire, which synchronously scores the five most bothersome post-proctectomy symptoms, was utilized in one case-matched study and as an adjunct in another study that did not necessarily focused on bowel dysfunction as a primary endpoint [10, 11]. In the Hain et. al case-matched study, LARS score was clearly inferior on the AL patients, but more relevant studies utilizing this globally accepted post-rectal resection patients’ interview tool are undoubtfully necessary [10]. LARS syndrome is quite common postoperatively but still underrecognized by clinicians even though that half or even more of the patients will face some degree of LARS symptoms; results that published by McKenna et. al. in their survey study in nearly 800 consecutive patients following rectal cancer surgery. [34] Nonetheless, this bowel function disorder can be improved over time, specifically 18 months after surgery as delineated in a Varghese et. al. recent meta-analysis [35].

Although urinary frequency, reduced male sexual activity and female dyspareunia may be linked to a prior AL, the limited number of under-powered studies, reaching even to contradictory results, impose the need for further targeted studies to counteract the existing literature vacuum [9, 10, 14].

Only Miura et al. who used a specific faecal incontinence-related QoL questionnaire has not concluded to a positive correlation of the AL occurrence and the QoL impairment [13]. Emotional and physical function difficulties arose as predominant discriminators between the AL and the non-AL patients throughout the first year, according to our Greek study [15]. Our results were reinforced by Ashburn et al. who had stated that AL patients had exhibited worse physical and mental function 6 months up to 3 years after surgery [8]. The persistence of QoL disturbances beyond the immediate postoperative period may be explained by the fact that an adverse event such as the occurrence of an AL, which if successfully managed does influence QoL long-termly [15]. As a negative treatment experience, AL may result in reduced QoL estimated that may exceed beyond the incident timing [8, 25]. According to Ashburn et. al, worse patients’ worse QoL had paralleled the times of compromised bowel function, speculating that AL had been the common denominator negatively affecting both parameters [8]. Nowadays, functional results and QoL restoration are regarded important surgical outcome measures. Related data may assist in identifying a vulnerable group of patients at risk, such as the ones having experienced an AL, who might require more intensive follow-up and the expertise of ancillary supportive services, whereas relevant information may be offered at the preoperative detailed rectal cancer patients’ counselling. Quality of life questionnaires are also offered to elderly patients above 70 years of age in a Dutch study by Ketelaers et.al. This study showed that LARS can severely affect their quality of life and it is of paramount importance to describe preoperatively and thoroughly possible bowel dysfunction in their decision making process [36].

Finally, it is worth mentioning at least briefly three important topics in rectal cancer approach. The recent retrospective analysis of Siragusa et.al. which highlighted the importance of rectal cancer cases centralization as high volume units registered less complications postoperatively including anastomotic leak rate and the preoperative neoadjuvant chemoradiotherapy which despite its beneficial role, contributes to increased AL rates interestingly to those who had complete response as described in Zaborowski et. al. systematic review recently [37, 38]. Lately number of released studies bring to our attention that AL rates may be reduced through transanal approach for low and middle rectal cancer patients [39].

As evidenced by this literature review, the limited reports on this intriguing topic may trigger the initiative for planning and undertaking larger, multicentre studies on rectal cancer patients with varying degrees of AL severity. Focusing on all QoL functional dimensions with the aid of specialized questionnaires and validating tools may further delineate fine aspects of disturbances and would further assist at instituting guiding principles for clinical management of all AL-related adverse sequelae. Heterogeneity, number of studies and variety at methodology and severity of AL precluded the application of a formal meta-analytic methodology at the current review. Robust recommendations drawn from high-leveled evidence are still lacking. Nevertheless, meaningful conclusions on the potential deleterious functional sequelae of an AL may still be derived and may constitute a valuable aid when counseling patients with complicated postoperative course or when a rehabilitative effort is attempted at a multimodality colorectal unit.

## References

[1] Lu ZR, Rajendran N, Lynch AC, Heriot AG, Warrier SK (2016). Anastomotic leaks after restorative resections for rectal cancer compromise cancer outcomes and survival. Dis Colon Rectum.

[2] Slim K, Nini E, Forestier D, Kwiatkowski F, Panis Y, Chipponi J (2003). Methodological index for non-randomized studies (minors): development and validation of a new instrument. ANZ J Surg.

[3] Hallböök O, Sjödahl R (1996). Anastomotic leakage and functional outcome after anterior resection of the rectum. Br J Surg.

[4] Nesbakken A, Nygaard K, Lunde OC (2001). Outcome and late functional results after anastomotic leakage following mesorectal excision for rectal cancer. Br J Surg.

[5] Bittorf B, Stadelmaier U, Merkel S, Hohenberger W, Matzel KE (2003). Does anastomotic leakage affect functional outcome after rectal resection for cancer?. Langenbecks Arch Surg.

[6] Lim M, Akhtar S, Sasapu K, Harris K, Burke D, Sagar P, Finan P (2006). Clinical and subclinical leaks after low colorectal anastomosis: a clinical and radiologic study. Dis Colon Rectum.

[7] Riss S, Stremitzer S, Riss K, Mittlböck M, Bergmann M, Stift A (2011). Pelvic organ function and quality of life after anastomotic leakage following rectal cancer surgery. Wien Klin Wochenschr.

[8] Ashburn JH, Stocchi L, Kiran RP, Dietz DW, Remzi FH (2013). Consequences of anastomotic leak after restorative proctectomy for cancer: effect on long-term function and quality of life. Dis Colon Rectum.

[9] Mongin C, Maggiori L, Agostini J, Ferron M, Panis Y (2014). Does anastomotic leakage impair functional results and quality of life after laparoscopic sphincter-saving total mesorectal excision for rectal cancer? A case-matched study. Int J Colorectal Dis.

[10] Hain E, Manceau G, Maggiori L, Mongin C, la Denise JPA, Panis Y (2017). Bowel dysfunction after anastomotic leakage in laparoscopic sphincter-saving operative intervention for rectal cancer: a case-matched study in 46 patients using the Low Anterior Resection Score. Surgery.

[11] Hughes DL, Cornish J, Morris C (2017). Functional outcome following rectal surgery—predisposing factors for low anterior resection syndrome. Int J Colorectal Dis.

[12] Yokota M, Ito M, Nishizawa Y, Kobayashi A, Saito N (2017). The impact of anastomotic leakage on anal function following intersphincteric resection. World J Surg.

[13] Miura T, Sakamoto Y, Morohashi H, Yoshida T, Sato K, Hakamada K (2017). Risk factor for permanent stoma and incontinence quality of life after sphincter-preserving surgery for low rectal cancer without a diverting stoma. Ann Gastroenterol Surg.

[14] Hultberg DK, Svensson J, Jutesten H, Rutegård J, Matthiessen P, Lydrup ML, Rutegård M (2020). The impact of anastomotic leakage on long-term function after anterior resection for rectal cancer. Dis Colon Rectum.

[15] Marinatou A, Theodoropoulos G, Karanika S, Karantanos T, Siakavellas S, Spyropoulos B, Toutouzas K, Zografos G (2014). Do anastomotic leaks impair postoperative health-related quality of life after rectal cancer surgery? A case-matched study. Dis Colon Rectum.

[16] Jorge MJN, Wexner SD (1993). Etiology and management of fecal incontinence. Dis Colon Rectum.

[17] Vaizey CJ, Carapeti E, Cahill JA, Kamm MA (1999). Prospective comparison of faecal incontinence grading systems. Gut.

[18] Agachan F, Chen T, Pfeifer J, Reissman P, Wexner SD (1996). A constipation scoring system to simplify evaluation and management of constipated patients. Dis Colon Rectum.

[19] Juul T, Ahlberg M, Biondo S, Emmertsen KJ, Espin E, Jimenez LM, Matzel KE, Palmer G, Sauermann A, Trenti L, Zhang W, Laurberg S, Christensen P (2014). International validation of the low anterior resection syndrome score. Ann Surg.

[20] Rosen C, Brown J, Heiman S, Leib R (2000). The female sexual function index (FSFI): a multidimensional self-report instrument for the assessment of female sexual function. J Sex Marital Therapy.

[21] Rosen RC, Riley A, Wagner G, Osterloh IH, Kirkpatrick J, Mishra A (1997). The international index of erectile function (IIEF): a multidimensional scale for assessment of erectile dysfunction. Urology.

[22] Whistance RN, Conroy T, Chie W, Costantini A, Sezer O, Koller M, Johnson CD, Pilkington SA, Arraras J, Ben-Josef E, Pullyblank AM, Fayers P, Blazeby JM (2009). Clinical and psychometric validation of the EORTC QLQ-CR29 questionnaire module to assess health-related quality of life in patients with colorectal cancer. Eur J Cancer.

[23] Niezgoda HE, Pater JL (1993). A validation study of the domains of the core EORTC quality of life questionnaire. Qual Life Res.

[24] King MT (1996). The interpretation of scores from the EORTC quality of life questionnaire QLQ-C30. Qual Life Res.

[25] Ware J, Kosinski M, Keller SD (1996). A 12-Item Short-Form Health Survey: construction of scales and preliminary tests of reliability and validity. Med Care.

[26] Ware JE, Sherbourne CD (1992). The MOS 36-item short-form health survey (SF-36). I. Conceptual framework and item selection. Med Care.

[27] Rockwood TH, Church JM, Fleshman JW, Kane RL, Mavrantonis C, Thorson AG, Wexner SD, Bliss D, Lowry AC (2000). Fecal incontinence quality of life scale. Dis Colon Rectum.

[28] Hashimoto H, Shiokawa H, Funahashi K, Saito N, Sawada T, Shirouzu K, Yamada K, Sugihara K, Watanabe T, Sugita A, Tsunoda A, Yamaguchi S, Teramoto T (2010). Development and validation of a modified fecal incontinence quality of life scale for Japanese patients after intersphincteric resection for very low rectal cancer. J Gastroenterol.

[29] Eypasch E, Williams JI, Wood-Dauphinee S, Ure BM, Schmulling C, Neugebauer E, Troidl H (1995). Gastrointestinal Quality of Life Index: development, validation and application of a new instrument. Br J Surg.

[30] Theodoropoulos GE, Papanikolaou IG, Karantanos T, Zografos G (2013). Post-colectomy assessment of gastrointestinal function: a prospective study on colorectal cancer patients. Tech Coloproctol.

[31] Ahmad NZ, Abbas MH, Khan SU, Parvaiz A (2021). A meta-analysis of the role of diverting ileostomy after rectal cancer surgery. Int J Colorectal Dis.

[32] Degiuli M, Collaborators from the Italian Society of Surgical Oncology Colorectal Cancer Network Collaborative Group (2021). Risk factors for anastomotic leakage after anterior resection for rectal cancer (RALAR study): a nationwide retrospective study of the Italian Society of Surgical Oncology Colorectal Cancer Network Collaborative Group. Colorectal Dis.

[33] Podda M, Coccolini F, Gerardi C, Castellini G, Wilson MSJ, Sartelli M, Pacella D, Catena F, Peltrini R, Bracale U, Pisanu A (2022). Early versus delayed defunctioning ileostomy closure after low anterior resection for rectal cancer: a meta-analysis and trial sequential analysis of safety and functional outcomes. Int J Colorectal Dis.

[34] McKenna NP, Bews KA, Yost KJ, Cima RR, Habermann EB (2022). Bowel dysfunction after low anterior resection for colorectal cancer: a frequent late effect of surgery infrequently treated. J Am Coll Surg.

[35] Varghese C, Wells CI, O'Grady G, Christensen P, Bissett IP, Keane C, Longitudinal LARS Group (2022). The longitudinal course of low-anterior resection syndrome: an individual patient meta-analysis. Ann Surg.

[36] Ketelaers SHJ, van Heinsbergen M, Orsini RG, Vogelaar FJ, Konsten JLM, Nieuwenhuijzen GAP, Rutten HJT, Burger JWA, Bloemen JG (2022). Functional bowel complaints and the impact on quality of life after colorectal cancer surgery in the elderly. Front Oncol.

[37] Siragusa L, Sensi B, Vinci D, Franceschilli M, Pathirannehalage Don C, Bagaglini G, Bellato V, Campanelli M, Sica GS (2021). Volume-outcome relationship in rectal cancer surgery. Discov Oncol.

[38] Zaborowski AM, Stakelum A, Winter DC (2021). Anastomotic leak risk in complete responders to neoadjuvant therapy for rectal cancer: a systematic review. Int J Colorectal Dis.

[39] Aubert M, Mege D, Panis Y (2020). Total mesorectal excision for low and middle rectal cancer: laparoscopic versus transanal approach-a meta-analysis. Surg Endosc.

